# Magnitude and trends in inequalities in healthcare‐seeking behavior for pneumonia and mortality rate among under‐five children in Bangladesh: Evidence from nationwide cross‐sectional survey 2007 to 2017

**DOI:** 10.1002/hsr2.1744

**Published:** 2023-12-06

**Authors:** Satyajit Kundu, Md Wahidur Rahman Nizum, Fahmida Fayeza, Syed Sharaf Ahmed Chowdhury, Jhantu Bakchi, Azaz Bin Sharif

**Affiliations:** ^1^ Global Health Institute North South University Dhaka Bangladesh; ^2^ Department of Public Health North South University Dhaka Bangladesh; ^3^ Department of Biochemistry and Food Analysis, Faculty of Nutrition and Food Science Patuakhali Science and Technology University Patuakhali Bangladesh; ^4^ Department of Public Health Nutrition Primeasia University Dhaka Bangladesh

**Keywords:** BDHS, healthcare‐seeking behavior, inequality, pneumonia, under‐five mortality

## Abstract

**Background and Aims:**

Bangladesh did not have enough evidence on the current estimates and trend in inequities in the under‐five mortality rate (U5MR). There is also a shortage of evidence on trends and inequalities in healthcare‐seeking for pneumonia among under‐five children (U5C) in Bangladesh. Hence, this study investigated the inequalities in U5MR and health care seeking for pneumonia in U5C through socioeconomic and geographic disparities in Bangladesh between 2007 and 2017.

**Methods:**

Data from 2007, 2011, 2014, and 2017 Bangladesh Demographic and Health surveys were analyzed using the Health Equity Assessment Toolkit (HEAT) software by World Health Organization (WHO). The data on U5MR and healthcare‐seeking for pneumonia were first disaggregated into five equity dimensions: wealth status, education, child sex, place of residence, and administrative divisions. Second, using summary metrics such as difference (D), population attributable risk (PAR), ratio (R), and population attributable fraction (PAF), inequalities were assessed.

**Results:**

The U5MR declined from 73.9 deaths per 1000 live births in 2007 to 48.6 deaths in 2017, while the prevalence of healthcare‐seeking for pneumonia in U5C fluctuated over time (34.6% in 2007, 35.4% in 2011, 42.0% in 2014, and 39.8% in 2017). Profound socioeconomic and geographic disparities in U5MR and the prevalence of healthcare‐seeking for pneumonia in U5C favored the wealthy, educated, and urban residents. At the same time, the Sylhet division showed the worst situation for U5MR. There were also sex‐related disparities in U5MR (PAR = −4.5, 95% confidence interval: −5.3 to −3.7) with higher risk among male children than females.

**Conclusion:**

These results indicate that improving disadvantaged women, such as the poor, uneducated, and rural inhabitants, who exhibit disproportionate disparities in U5MR and healthcare‐seeking behavior is important. To reduce childhood mortality, it is essential to improve healthcare‐seeking for pneumonia among U5C. Facilitating women for better education and economic encompasses would help reducing disparity.

## 1 INTRODUCTION

Child mortality is a serious public health concern and a key metric for gauging a nation's development. Globally, 16,000 children die daily, with 11 deaths every minute.^1^ South Asian countries account for three out of 10 global child fatalities. The majority of under‐five mortality (U5M) is made up of neonatal (the first 28 days of life) and infant (the first year of life) deaths in South Asia^2^ According to the Bangladesh Demographic and Health Survey (BDHS), in 2014, 46 deaths per 1000 live births were recorded for under‐five children (U5C);^3^ in 2017, 45 deaths per 1000 live births were recorded.^4^

Despite Bangladesh's significant advancements in improving maternal under‐nutrition, reduction in adolescent pregnancy, increase in breastfeeding practices, growth in immunization, and rise in the use of maternal healthcare services, the country exhibits a much higher rate of child mortality compared with other South Asian countries like Sri Lanka, Nepal, Bhutan and Maldives.^5^, ^6^, ^7^ Consequently, Bangladesh and most of the low‐ and middle‐income (LMIC) countries are falling short of the Sustainable Development Goals (SDGs') for reducing child mortality.^8^ Among the top 10 diseases that contribute to the higher prevalence of U5M globally, pneumonia holds the position in the first quintile.^9^ Pneumonia is one of the prime causes of mortality among U5C, accounting for 15% of all fatalities globally. The prevalence of pneumonia is around 10 times higher in low‐income countries than in high‐income countries.^10^ As of 2016, 1.87 million new cases of pneumonia were detected annually, with Bangladesh being one of the five nations that account for more than half of all pediatric pneumonia cases worldwide.^9^, ^11^

Despite high childhood mortality and morbidity rates, Bangladeshi mothers manifested notably low healthcare‐seeking behavior for ill U5C.^12^ Previous research showed that traditional geographic and financial barriers^13^ to care, as well as a lack of awareness of maternal and infant danger signs,^14^ can cause delays in receiving timely medical attention from skilled professionals for pneumonia.^15^ Proper treatment from professionals with medical training and adequately equipped healthcare facilities are crucial for reducing child mortality and morbidity.^16^ The SDG to eliminate preventable deaths of children under five by 2030 is particularly hampered by the inadequate usage of healthcare services.^17^ Previous studies reported that the delayed decline in child mortality was thought to be influenced by socioeconomic status, especially in developing countries.^18^ Regardless of the level of development, the gap due to the socioeconomic status in child health and mortality has been troubling for many countries, including Bangladesh.^19^, ^20^ Even though many public health services, including child health care, are free of charge, the poor have lesser access to health care than those who are better affluent because poor people face social and cultural hurdles and are less educated.^21^

A study from Bangladesh identified that the leading cause of mortality for children under five in Bangladesh is pneumonia, which accounts for around 19% of annual fatalities.^22^ It suggests that the mortality due to pneumonia should be curved to reduce the overall U5MR in Bangladesh. However, the inequality in health concerns has recently garnered increased attention internationally with its explicit mention as a development objective in the global agenda, such as the SDGs.^23^ The best way to reduce the inequalities to a manageable level remained a mystery. Therefore, it has become crucial to know the helm of both socioeconomic and geographic inequalities in U5M and healthcare‐seeking behavior for pneumonia to design target‐based and site‐specific interventions. Though very few studies have assessed the U5M issue in Bangladesh, like time, place, and causes of mortality,^22^ and determinants,^24^ there is a lack of studies that looked at the systematic and comprehensive investigation of inequalities in U5MR and healthcare‐seeking behavior for pneumonia among U5C in Bangladesh.

Therefore, this study aims to investigate the magnitude and patterns in inequalities in U5MR and health care seeking for pneumonia in U5C based on socioeconomic and geographical dimensions in Bangladesh between 2007 and 2017.

## 2 METHODS

### 2.1 Study design and data source

To conduct this study, we utilized Bangladesh Demographic and Health Survey data from 2007, 2011, 2014, and 2017–2018. The BDHS is a component of the international surveys that conduct Demographic and Health Surveys (DHS) in 90 LMICs. Using a cross‐sectional design, DHS's main objective is to compile and collect data regarding demographic and health information of men, women, and children. To collect nationally representative data, DHS employs a two‐stage cluster sampling approach.^25^, ^26^ In partnership with USAID, the National Institute of Population Research and Training (NIPORT) and the Ministry of Health and Family Welfare of Bangladesh conduct the BDHS. Details about the ethical guidelines, methodologies, sampling techniques, and survey instruments used in BDHS 2007, 2011, 2014, and 2017–2018 are outlined elsewhere.^3^, ^4^, ^27^, ^28^ All data from these four waves of BDHS were deposited in the WHO Health Equity Assessment Toolkit (HEAT) software^29^ for analysis.

### 2.2 Outcome variables

Healthcare‐seeking behavior for pneumonia among U5C and U5MR were the two outcome variables of this study. Mothers were asked whether or not children under 5 years with pneumonia symptoms were taken to a health facility. The answer to this question had a dichotomized response as yes/no. U5MR was presented as the number of deaths per 1000 live births. The birth record data of BDHS (BR file) contain information on the birth date and age of death of the U5C.

### 2.3 Measures of inequality

The inequalities of healthcare‐seeking behavior for pneumonia among U5C and U5MR were measured using five inequality dimensions: household wealth status (quintiles), educational level, sex of the children, place of residence, and subnational regions. Data for both outcomes of this study were disaggregated by these five equity dimensions. The DHS uses the Principal Component Analysis (PCA) method to generate the wealth index utilizing household income, various household assets, and characteristics.^30^ We used the five‐quintile wealth index, categorized as poorest, poorer, middle, richer, and richest. The mother's educational level was classified as no education, primary education, secondary/higher education. The place of residence was categorized into rural and urban. Subnational regions were the administrative divisions of Bangladesh. For 2017–2018 data set, the subnational regions were Barishal, Chattogram, Dhaka, Khulna, Mymensingh, Rajshahi, Rangpur, and Sylhet, where Barishal, Chattogram, and Khulna are from the southern coastal region of Bangladesh, Dhaka is the capital and center of Bangladesh, Rajshahi and Rangpur are from the northern part of Bangladesh, and Mymensingh and Sylhet are from the northern‐east part of Bangladesh Rangpur division was separated from Rajshahi division in 2010, and Mymensingh division was separated from Dhaka division in 2015. Hence, the estimates for BDHS 2004–2014 data of Mymensingh and BDHS 2004–2007 data of Rangpur division are not shown in the tables.

### 2.4 Statistical analyses

Analyses were conducted using HEAT software (2022 update version 4.0) of the World Health Organization (WHO) using data from the reproductive, maternal, newborn, and child health datasets of the WHO Health Inequality Monitor data repository.^31^ First, the prevalence of healthcare‐seeking behavior for pneumonia among U5C and U5MR were disaggregated by the five equity dimensions. The disaggregation allowed us to present the distribution of the estimates and confidence intervals of healthcare‐seeking behavior for pneumonia among U5C and U5MR. Then, inequalities were assessed using four disparities measures: Difference, population attributable risk (PAR), population attributable fraction (PAF), and ratio. The difference and ratio are simple unweighted measures, while PAF and PAR are complex weighted measures. Alternatively, ratio and PAF are relative measures, while Difference and PAR are absolute measures. We estimated both absolute and relative measures because, according to the WHO, producing results that influence public policy requires using both absolute and relative summary metrics.^32^ Consequently, integrating both relative and absolute measures makes a study more thorough. Additional details on how to calculate these summary measurements are provided elsewhere.^32^, ^33^

A zero PAF and PAR value indicates no inequality, whereas a larger absolute PAF and PAR values indicate a greater degree of disparity. The difference between the subgroup with the lowest estimate and the national average of the indicator for unfavorable outcomes was used to construct the PAR estimate.^34^ Regardless of the indicator type, the difference and ratio were estimated between the subgroups with the highest estimate (e.g., the richest wealth quintile) and the lowest estimate (e.g., the poorest wealth quintile). When the difference and ratio values were 0 and 1, respectively, we assumed that inequality was absent. We calculated 95% confidence intervals (CIs) around point estimates of each measure for each survey wave to evaluate if U5MR and healthcare‐seeking behavior for pneumonia in U5C show significant inequalities across the subgroups of each equity dimension. The lower and upper bounds of the CI must not include 0 for any inequality measure other than Ratio to conclude that an inequality exists. For ratio, the interval should not contain one to conclude that an inequality exists.^35^ All the statistical tests to determine the estimates and their significance were two‐sided.

### 2.5 Ethical consideration

The study used deidentified data from the Demographic Health Survey program, which has already received ethical approval from the participating countries; no further ethical permission was sought to carry out this research. Data was collected from an online source (https://dhsprogram.com) with an appropriate request. Written informed consent from the respondents enrolled in the survey and other ethical review documents are available at: https://dhsprogram.com/methodology/Protecting-the-Privacy-of-DHS-Survey-Respondents.cfm. The data set is available online publicly for all researchers; hence there is no need to approve.

## 3 RESULTS

### 3.1 Distribution of under‐five mortality rate (U5MR) across different subgroups

**Figure 1:**
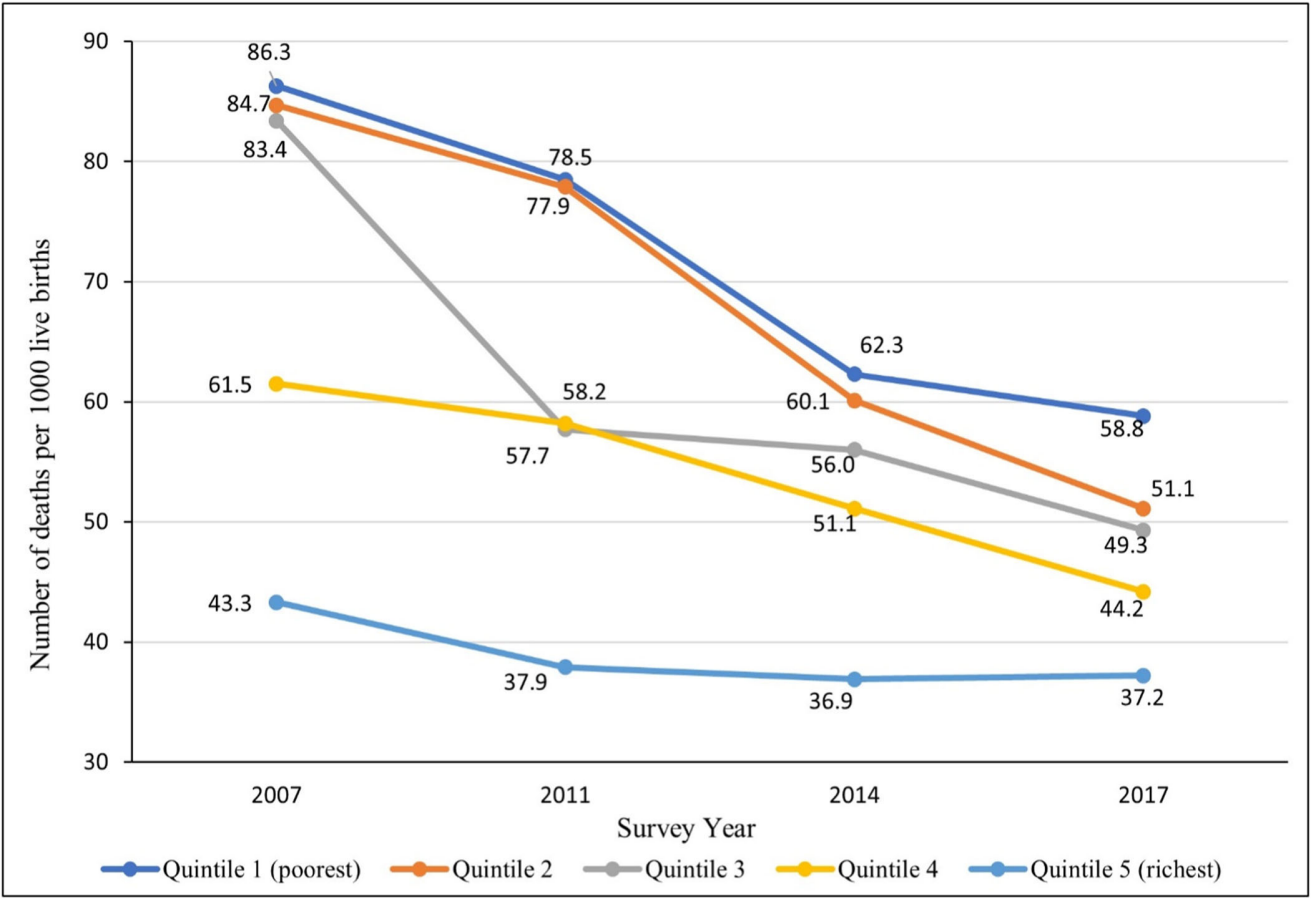
U5MR in Bangladesh by wealth quintiles: evidence from BDHS (2007–2017). BDHS, Bangladesh Demographic and Health Survey; U5MR, under‐five mortality rate.

**Figure 2:**
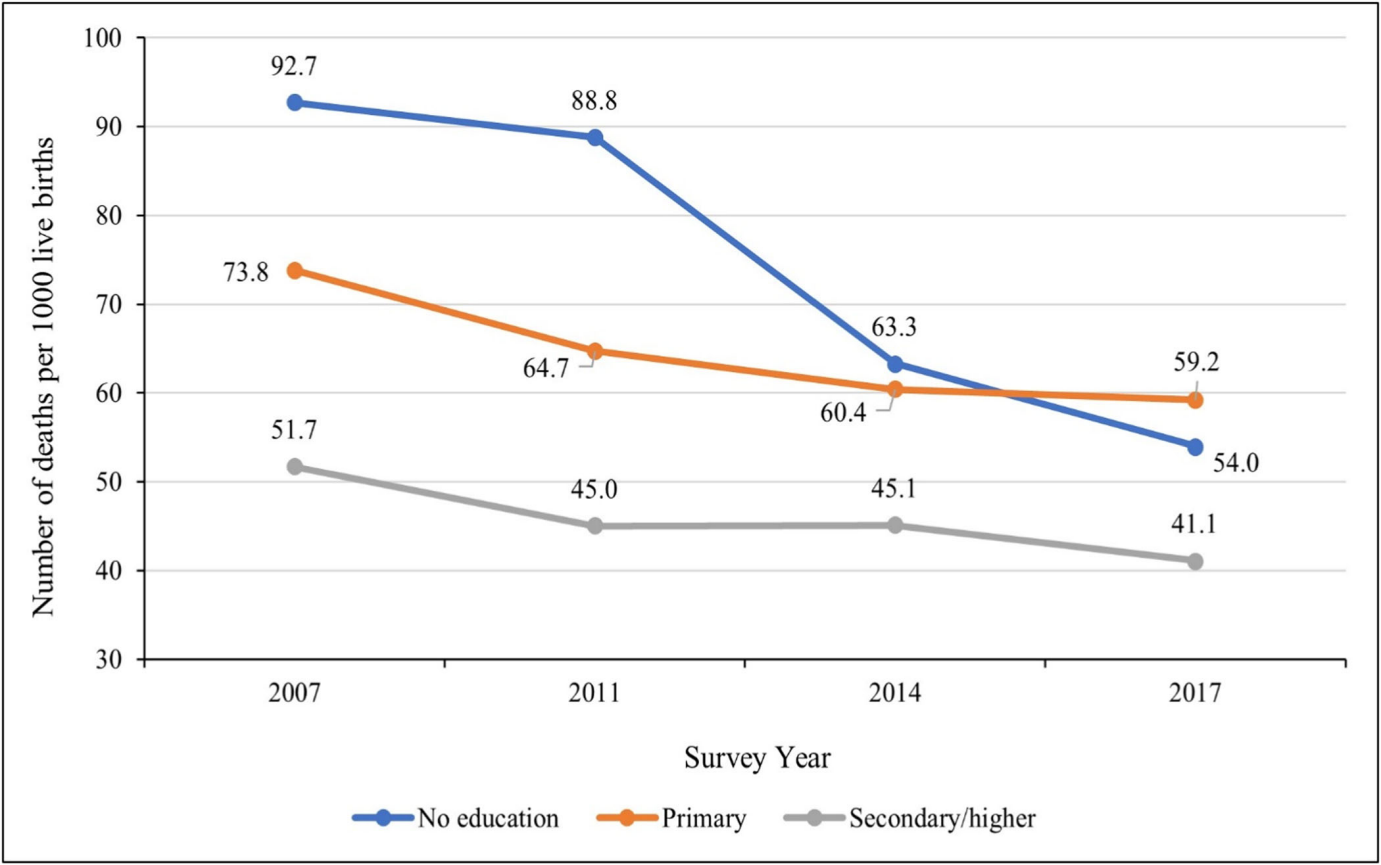
U5MR in Bangladesh by educational status of mothers: evidence from BDHS (2007–2017). BDHS, Bangladesh Demographic and Health Survey; U5MR, under‐five mortality rate.

Figure 1 shows the trend of U5MR among socioeconomic subgroups from 2007 to 2017 in Bangladesh. U5MR was higher among the poorest (wealth quantile 1) group by 43 deaths per 1000 live births in 2007, 40.6 in 2011, 25.4 in 2014, and 20.8 in 2017 than among the richest (wealth quantile 5) population. The U5MR was also found to be higher among the mothers with no formal education than the mothers who had completed secondary or higher education. In 2007, U5MR was higher by 41 per 1000 live births, by 43.8 in 2011, by 18.2 in 2014, and by 12.9 in 2017 among mothers without institutional education (Figure 2).

The overall U5MR was 73.9 per 1000 live births in 2007, 63.6 in 2011, 54 in 2014, and 48.6 in 2017. Regarding child sex, the U5MR was higher by 3.4 deaths per 1000 live births among male children in 2007, 5.8 in 2011, 10.1 in 2014, and 2.5 in 2017, compared to female children. The U5MR was higher by 13.7 per 1000 live births in 2007 among rural children compared to the children who resided in the urban area, and the pattern was consistent in the subsequent survey years (Table 1).

**Table 1:** Children under five mortality rates across socioeconomic and geographic subpopulations in Bangladesh, disaggregated across five inequality dimensions, 2004–2017.

Inequality dimension	2007 (73.9)		2011 (63.6)		2014 (54.0)		2017 (48.6)	
	*N*	Estimate (95% CI)	*N*	Estimate (95% CI)	*N*	Estimate (95% CI)	*N*	Estimate (95% CI)
Wealth status								
Quintile 1 (poorest)	3023	86.3 (70.8–101.9)	4532	78.5 (69.5–87.5)	4062	62.3 (52.0–72.5)	3998	58.8 (49.8–67.7)
Quintile 2	2735	84.7 (70.3–99.1)	3782	77.9 (67.8–88.0)	3417	60.1 (48.0–72.2)	3580	51.1 (41.9–60.4)
Quintile 3	2459	83.4 (71.6–95.1)	3540	57.7 (48.3–67.0)	3294	56.0 (41.0–71.1)	3246	49.3 (40.6–57.9)
Quintile 4	2234	61.5 (49.4–73.6)	3487	58.2 (48.0–68.5)	3216	51.1 (40.8–61.3)	3491	44.2 (35.6–52.8)
Quintile 5 (richest)	2116	43.3 (33.2–53.3)	3226	37.9 (30.1–45.8)	3072	36.9 (28.0–45.8)	3265	37.2 (28.7–45.6)
Education level								
No education	4406	92.7 (79.7–105.8)	4846	88.8 (78.3–99.4)	3764	63.3 (52.0–74.6)	1924	54.0 (42.8–65.2)
Primary	3954	73.8 (63.5–84.1)	6009	64.7 (57.5–71.9)	5080	60.4 (51.3–69.4)	5608	59.2 (51.7–66.7)
Secondary/higher	4181	51.7 (42.8–60.6)	7711	45.0 (39.3–50.6)	8218	45.1 (39.4–50.7)	10048	41.1 (36.5–45.7)
Child sex								
Male	6357	75.5 (67.8–83.2)	9544	66.4 (60.0–72.8)	8797	52.2 (46.7–57.8)	9074	52.8 (47.5–58.2)
Female	6210	72.1 (63.0–81.3)	9022	60.6 (54.4–66.7)	8265	55.8 (48.4–63.2)	8506	44.1 (38.9–49.4)
Place of residence								
Rural	10016	76.6 (68.5–84.7)	14417	66.0 (60.8–71.2)	12745	56.5 (51.2–61.7)	12778	49.3 (44.9–53.8)
Urban	2551	62.9 (53.7–72.2)	4150	55.2 (46.2–64.3)	4317	46.4 (38.5–54.4)	4802	46.8 (39.5–54.1)
Subnational regions								
Barishal	804	71.3 (59.9–82.7)	1012	62.2 (51.7–72.7)	991	51.9 (41.7–62.0)	968	51.0 (40.9–61.0)
Chattogram	2640	79.4 (65.2–93.6)	4068	63.3 (52.0–74.6)	3673	61.7 (50.4–73.0)	3680	45.8 (36.1–55.5)
Dhaka	4000	68.6 (55.1–82.1)	5902	62.5 (53.0–71.9)	5920	47.5 (39.2–55.8)	4447	47.7 (38.4–57.0)
Khulna	1266	58.1 (44.1–72.0)	1725	47.3 (39–55.7)	1346	50.4 (40.3–60.5)	1634	38.2 (29.1–47.2)
Mymensingh	‐	‐	‐	‐	‐	‐	1447	51.2 (42.1–60.3)
Rajshahi	2749	71.0 (54.5–87.5)	2418	73.7 (60.9–86.4)	1721	50.1 (40.1–60.2)	2064	52.0 (42.2–61.8)
Rangpur	‐	‐	2029	59.0 (48.0–70.0)	1698	45.1 (32.3–57.9)	1923	47.5 (36.1–58.9)
Sylhet	1108	107.4 (84.3–130.4)	1412	80.2 (69.0–91.4)	1713	76.8 (66.3–87.2)	1418	63.4 (53.7–73.1)

*Note*: Mymensingh division was separated from Dhaka division in 2015, and Rangpur division was separated from Rajshahi division in 2010. Hence, the estimates for BDHS 2004–2014 data of Mymensingh, and BDHS 2004–2007 data of Rangpur division are not shown in the table.

Abbreviations: BDHS, Bangladesh Demographic and Health Survey; CI, confidence interval.

### 3.2 Magnitude and trends in disparities in U5MR

Table 2 represents the socioeconomic, educational, gender, urban‐rural, and sub‐regional inequalities in U5MR in Bangladesh from 2007 to 2017. The results showed that disadvantaged groups had a higher burden of U5MRs over the years than socioeconomically and geographically advantaged populations. Over the past decade, we identified wealth‐driven disparities in the U5MR by both simple (D) and complex (PAR and PAF) measures, with a greater concentration among disadvantaged subpopulations, such as the poorest populations, compared to the richest. For example, the PAF measure in 2017 (−23.5, 95% CI: −26.6 to −20.4) indicated wealth‐related inequality with a greater burden on the poorest subpopulation. From 2007 to 2017, using all four summary measures (D, PAF, PAR, and R), this study showed a higher burden among the non‐educated subpopulations. For instance, in the 2017 survey, the PAF and PAR measures of −14.9 (95% CI: −16.2 to −13.5) and −7.2 (95% CI: −7.8 to −6.5) indicated significant education‐related disparities in U5M with higher burden among children of mothers having no formal education. Furthermore, the study also identified sex‐related absolute and relative disparities in U5MR with higher concentration among male children compared to females. For example, in the most recent survey of 2017, the PAF and PAR measures of −9.2 (95% CI: −10.8 to −7.7) and −4.5 (95% CI: −5.3 to −3.7) indicated that wide disparities in U5MR through the sex subgroups, favoring female children. Besides, the study also showed pro‐urban absolute and relative inequalities in U5M from 2007 to 2017. For example, the measures of PAF (−3.7, 95% CI: −6.2 to −1.3) and PAR (−1.8, 95% CI: −3 to −0.6) in 2017 demonstrated that rural–urban disparities found in U5M while disfavoring the children from the rural area. The study also found geographical disparities in U5MR from 2007 to 2017. The PAF and PAR measures of −21.5 (95% CI: −26.1 to −16.9) and −10.5 (95% CI: −12.7 to −8.2), respectively, in 2017 showed that absolute and relative geographical inequalities in U5M with higher burden in Sylhet division.

**Table 2:** Inequality indices estimates of the factors associated with under 5 children mortality rate in Bangladesh, 2004–2017.

Inequality dimension	2007		2011		2014		2017	
	Estimate	95% CI	Estimate	95% CI	Estimate	95% CI	Estimate	95% CI
Wealth status								
Difference	43.0	24.6, 61.5	40.6	28.7, 52.5	25.3	11.8, 38.8	21.6	9.4, 33.9
PAF	−41.3	−44.0, −38.7	−40.3	−42.8, −37.9	−31.6	−34.5, −28.7	−23.5	−26.6, −20.4
PAR	−30.5	−32.4, −28.5	−25.6	−27.2, −24.1	−17.0	−18.6, −15.5	−11.4	−12.9, −9.9
Ratio	2.0	1.5, 2.7	2.1	1.6, 2.6	1.7	1.3, 2.3	1.6	1.2, 2.1
Education level								
Difference	41.0	25.3, 56.8	43.9	31.9, 55.8	18.2	5.6, 30.8	12.9	0.8, 25
PAF	−29.3	−31.0, −27.6	−28.4	−29.8, −27	−16.0	−17.4, −14.5	−14.9	−16.2, −13.5
PAR	−21.4	−22.6, −20.2	−17.8	−18.7, −17	−8.6	−9.4, −7.8	−7.2	−7.8, −6.5
Ratio	1.8	1.4, 2.2	2.0	1.7, 2.3	1.4	1.1, 1.7	1.3	1.0, 1.7
Child sex								
Difference	3.4	−8.5, 15.3	5.8	−3.0, 14.7	−3.5	−12.8, 5.7	8.7	1.2, 16.2
PAF	−2.3	−3.4, −1.3	−4.7	−5.9, −3.6	0.0	−1.4, 1.4	−9.2	−10.8, −7.7
PAR	−1.7	−2.5, −0.9	−3.0	−3.7, −2.3	0.0	−0.8, 0.8	−4.5	−5.3, −3.7
Ratio	1.0	0.9, 1.2	1.1	1.0, 1.3	0.9	0.8, 1.1	1.2	1.0, 1.4
Place of residence								
Difference	13.7	1.4, 25.9	10.8	0.4, 21.1	10.1	0.6, 19.6	2.5	−6, 11.0
PAF	−14.7	−17, −12.5	−13.1	−15.2, −11.1	−13.9	−16.3, −11.6	−3.7	−6.2, −1.3
PAR	−10.9	−12.5, −9.2	−8.4	−9.7, −7.0	−7.5	−8.8, −6.2	−1.8	−3.0, −0.6
Ratio	1.2	1.0, 1.5	1.2	1.0, 1.4	1.2	1.0, 1.5	1.1	0.9, 1.3
Subnational regions								
Difference	49.3	23.0, 75.6	32.8	19.1, 46.6	31.7	15.4, 48	25.2	12.1, 38.3
PAF	−21.4	−24.9, −18.0	−25.6	−29.1, −22.1	−16.5	−20.7, −12.4	−21.5	−26.1, −16.9
PAR	−15.9	−18.4, −13.3	−16.3	−18.5, −14.1	−8.9	−11.2, −6.7	−10.5	−12.7, −8.2
Ratio	1.8	1.4, 2.5	1.7	1.4, 2.1	1.7	1.2, 2.3	1.7	1.3, 2.2

*Note*: Difference and Ratio are relative measures, while PAR and PAF are absolute summary measures.

Abbreviations: CI, confidence interval; PAF, population attributable fraction; PAR, population attributable risk.

### 3.3 Distribution of prevalence of health care seeking for pneumonia among U5C across different subgroups

Table 3 represents the prevalence of health facilities use for U5C with pneumonia symptoms across different population subgroups. The trends in using health facilities for pneumonia symptoms have increased over the years, except in 2017; the prevalence was 34.6% in 2007, 35.4% in 2011, 42.0% in 2014, and 39.8% in 2017. The use of health facilities for pneumonia symptoms among the population from the richest wealth quantile was relatively higher than those from the poorest wealth quantile over the years. The usage of health facilities was higher by 47.6 percentage points in 2007, 34 percentage points in 2011, 22 percentage points in 2014, and 19.4 percentage points in 2017 among the richest group. A similar pattern was also observed among the children whose mothers completed secondary or higher education compared to those without formal education. When looking at the child sex, in 2007 and 2017, healthcare facility use was higher by 6.6 and 14.4 percentage points among male children. On the contrary, the usage was higher by 24 and 7.9 percentage points among female children in 2011 and 2014. Healthcare facility use was also higher among children from urban areas in all survey waves except in 2011 compared to those from rural areas. There was a variation in using health facilities for child pneumonia symptoms across subnational regions. For example, it was higher in the Barishal division compared to other divisions in 2007 and 2017, while it was higher in Rangpur division in 2011 and Chattogram in 2014.

**Table 3:** Trends in prevalence of health facility use for children under 5 years with pneumonia symptoms, disaggregated across five inequality dimensions, 2007–2017.

Inequality dimension	2007 (34.6%)		2011 (35.4%)		2014 (42.0%)		2017‐18 (39.8%)	
	*N*	Estimate (95% CI)	*N*	Estimate (95% CI)	*N*	Estimate (95% CI)	*N*	Estimate (95% CI)
Wealth status								
Quintile 1 (poorest)	83	23.0 (13.6–36.3)	143	24.7 (18.0–32.8)	116	37.8 (22.0–56.7)	80	35.0 (25.4–45.9)
Quintile 2	71	26.3 (15.9–40.4)	92	30.3 (21.3–41.1)	92	45.4 (33.1–58.4)	56	35.1 (22.2–50.5)
Quintile 3	44	38.4 (22.9–56.6)	97	29.1 (20.2–40.0)	79	36.0 (25.0–48.6)	40	41.9 (26.6–58.9)
Quintile 4	52	43.0 (29.0–58.2)	77	46.2 (34.3–58.6)	80	38.8 (26.0–53.4)	46	42.1 (27.6–58.0)
Quintile 5 (richest)	26	70.6 (51.0–84.7)	76	58.7 (43.7–72.2)	50	59.8 (44.2–73.6)	32	54.4 (36.1–71.7)
Education level								
No education	81	26.0 (16.3–38.8)	116	25.4 (18.1–34.5)	57	31.2 (18.9–47.0)	11	‐
Primary	92	34.4 (23.0–47.9)	154	29.7 (22.2–38.3)	156	41.1 (27.5–56.2)	86	25.5 (17.4–35.7)
Secondary/higher	102	42.1 (30.0–55.2)	216	44.8 (37.0–52.9)	203	45.7 (36.8–54.8)	157	47.1 (38.8–55.6)
Child sex								
Male	149	37.7 (28.5–47.8)	397	31.0 (25.9–36.6)	248	38.8 (29.1–49.5)	159	45.2 (37.1–53.4)
Female	128	31.1 (22.7–40.9)	89	55.0 (44.2–65.3)	169	46.7 (37.0–56.5)	95	30.8 (22.1–41.0)
Place of residence								
Rural	237	31.5 (24.3–39.7)	281	38.8 (32.7–45.3)	331	39.3 (31.3–48.0)	193	38.2 (31.3–45.5)
Urban	40	53.2 (36.8–69.0)	205	30.7 (24.2–38.0)	86	52.1 (38.9–65.0)	61	44.9 (31.9–58.7)
Subnational regions								
Barishal	17	40.1 (19.3–65.3)	33	40.1 (27.6–54.1)	18	38.6 (25.3–53.9)	20	56.2 (41.0–70.3)
Chattogram	68	33.4 (21.0–48.6)	144	26.3 (18.7–35.7)	81	46.3 (34.1–59.0)	48	50.0 (32.5–67.4)
Dhaka	68	37.1 (23.7–52.9)	121	36.5 (25.9–48.6)	141	43.2 (27.3–60.7)	45	25.4 (12.5–44.8)
Khulna	21	‐	49	45.4 (33.9–57.4)	35	44.8 (32.8–57.5)	13	‐
Mymensingh	‐	‐	‐	‐	‐	‐	17	‐
Rajshahi	71	30.9 (17.5–48.6)	59	31.1 (19.3–46.0)	53	37.3 (25.3–51.1)	38	35.3 (21.7–51.7)
Rangpur			48	46.6 (32.1–61.7)	40	30.1 (17.7–46.2)	53	42.5 (30.3–55.6)
Sylhet	31	31.4 (21.3–43.5)	32	43.2 (29.8–57.6)	49	45.0 (29.3–61.8)	20	41.3 (24.6–60.4)

*Note*: Mymensingh division was separated from Dhaka division in 2015, and Rangpur division was separated from Rajshahi division in 2010. Hence, the estimates for BDHS 2004–2014 data of Mymensingh, and BDHS 2004–2007 data of Rangpur division are not shown in the table

Abbreviations: BDHS, Bangladesh Demographic and Health Survey; CI, confidence interval.

### 3.4 Magnitude and trends in disparities in healthcare seeking for pneumonia symptoms

Table 4 represents inequalities in accessing health facilities for U5C with pneumonia symptoms in Bangladesh from 2007 to 2017 by socioeconomic status, educational level, gender, urban–rural, and subnational regions. The results showed disparities in using health facilities for pneumonia symptoms, favoring the economically advantaged groups compared to the economically disadvantaged groups. For example, the PAF measure of 36.7 (95% CI: 14.6–58.9) in 2017 indicates higher usage of health facilities among the richest subgroup, highlighting the wealth‐driven disparities. Similarly, we found significant education‐related inequalities in 2011 that disfavored the non‐educated population. To be more specific, the PAR measures (9.4, 95% CI: 2.2–16.7) in 2011 suggest that education‐related inequalities in using health facilities favor the educated subgroup. Furthermore, this study shows absolute rural–urban disparities in using healthcare facilities. For example, the PAF measures of 12.9 (95% CI: 4.3–21.5) in 2017 indicate significant pro‐urban disparities in healthcare facility utilization.

**Table 4:** Inequality indices estimates of the factors associated with health facility use for children under 5 years with pneumonia symptoms, 2007–2017.

Inequality dimension	2007		2011		2014		2017	
	Estimate	95% CI	Estimate	95% CI	Estimate	95% CI	Estimate	95% CI
Wealth status								
Difference	47.6	27.3–67.9	34.0	17.6–50.4	22.0	−1.4, 45.4	19.4	−1.7, 40.6
PAF	104.0	80.5–127.5	65.8	48.1–83.4	42.4	24.3–60.6	36.7	14.6–58.9
PAR	36.0	27.9–44.1	23.3	17.0–29.5	17.8	10.2–25.4	14.6	5.8–23.5
Ratio	3.1	1.8–5.3	2.4	1.6–3.5	1.6	0.9–2.7	1.6	1.0–2.4
Education level								
Difference	16.1	−0.7, 32.8	19.4	7.9–30.8	14.4	−2.5, 31.4	‐	‐
PAF	21.0	−3.3, 45.3	26.7	6.3–47.1	8.8	−18.7, 36.3	‐	‐
PAR	7.3	−1.2, 15.8	9.4	2.2–16.7	3.7	−7.8, 15.2	‐	‐
Ratio	1.6	1.0–2.7	1.8	1.2–2.5	1.5	0.9–2.4	‐	‐
Child sex								
Difference	−6.6	−19.6, 6.5	−8.1	−17.5, 1.2	7.9	−6.3, 22.1	−14.4	−26.9, −1.9
PAF	0.0	−15.0, 15.0	0.0	−10.2, 10.2	11.2	1.8–20.6	0.0	−11.6, 11.6
PAR	0.0	−5.2, 5.2	0.0	−3.6, 3.6	4.7	0.7–8.6	0.0	−4.6, 4.6
Ratio	0.8	0.6–1.2	0.8	0.6–1.0	1.2	0.9–1.7	0.7	0.5–1.0
Place of residence								
Difference	21.7	3.7–39.6	24.0	12.1–36.0	12.7	−3.0, 28.5	6.8	−8.6, 22.1
PAF	53.6	46.3–61.0	55.4	49.1–61.7	24.1	18.2–30.1	12.9	4.3–21.5
PAR	18.6	16.0–21.1	19.6	17.4–21.8	10.1	7.6–12.6	5.1	1.7–8.6
Ratio	1.7	1.1–2.5	1.8	1.4–2.3	1.3	0.9–1.8	1.2	0.8–1.7
Subnational regions								
Difference	‐	‐	20.3	2.9–37.7	16.3	−3.0, 35.5	‐	‐
PAF	‐	‐	31.6	13.8–49.4	10.4	−23.3, 44.0	‐	‐
PAR	‐	‐	11.2	4.9–17.5	4.3	−9.8, 18.5	‐	‐
Ratio	‐	‐	1.8	1.1–2.8	1.5	0.9–2.7	‐	‐

*Note*: Difference and ratio are relative measures, while PAR and PAF are absolute summary measures.

Abbreviations: CI, confidence interval; PAF, population attributable fraction; PAR, population attributable risk.

## 4 DISCUSSION

This study aimed to measure the magnitude and trend of inequality in seeking health care for pneumonia and mortalities among U5C over time using the last four rounds of BDHS data. This study found inconsistently fluctuating inequalities in all dimensions over time. Inequalities in healthcare‐seeking behavior for pneumonia in children were found to have increased, while U5MR decreased in most of the dimensions over the survey period. The reduction in U5M can be explained by higher healthcare‐seeking behavior among the mothers of the children over time, leading to a lower prevalence of acute respiratory tract infection^36^ and an impressive improvement in neonatal mortality since pneumonia and neonatal mortality are the greatest contributors to U5M.^37^ Besides the reform in the health sector significantly covering reproductive, maternal, child, and neonatal health care access, better coverage by the health services can also be a possible reason behind these findings.^38^, ^39^

Despite the decrease in the U5M and increase in the care‐seeking behavior for pneumonia among the mothers of the U5C, a significant gap in the prevalence between the poorest and the richest group could be the contributing factor behind the inequalities. The decreasing pattern of U5M was also found in the studies conducted in Nigeria,^40^ Bangladesh,^41^ and other South Asian countries.^42^ The result can be explained by the wealthier subgroups having better access to health care, better education, and raised awareness on healthcare seeking compared with those from less wealthy families.^43^, ^44^ Again, wealthier women were found to have higher health‐seeking behavior for common childhood illnesses in Ethiopia,^45^ which can be a probable reason for decreased U5M among this subgroup. An increase in the use of health facilities for childhood illness with increasing wealth quintile was also found to be consistent with the studies conducted in Bangladesh,^46^ Ethiopia,^47^ and sub‐Saharan Africa.^48^ This might be because the decision to use the health facility is affected by the out‐of‐pocket expenditure, and in LMIC, like Bangladesh, wealthier subgroups are generally the only ones who can afford this.^49^, ^50^, ^51^ This might lead to lower health‐seeking behavior among the disadvantaged subgroups.

In our study, we found that both the U5M and health care seeking for pneumonia in U5C have persistent inequality in the dimension of the mother's level of education. Maternal education was found to have an inverse relationship with U5M and a forward relation with the care‐seeking behavior for pneumonia, with the higher‐educated subgroup being the advantageous and lower lower‐educated being the disfavored group. Lower U5M in more educated mothers was also reported in a meta‐analysis^52^ and some studies in LMIC countries.^53^, ^54^, ^55^ This pattern of result might be due to the fact that more educated women are better aware of their child's health problems and better informed about the availability of the health facility^44^ than the less educated women. Again, educated women tend to be more empowered in decision‐making, especially when seeking health care,^56^, ^57^ which might be another reason behind the increasing pattern of health care seeking for pneumonia leading to decreased U5M.

This study observed gender‐based disparities of U5MR in Bangladesh, comparable with a prior study conducted in Nigeria.^58^ Both studies had shown that the U5MR was relatively higher among male children than females. However, the gender‐based inequalities in healthcare‐seeking behavior for pneumonia fluctuated across the survey waves. Previous studies reported no or weak association between child sex and healthcare‐seeking behavior.^47^, ^59^ On the other hand, studies conducted in Nigeria and India found a significant association between these two variables.^59^ Variations in sample sizes, demography, and contextual factors between studies may explain these differences. Moreover, differences in gender‐specific health policies across countries could also contribute to this disparity.

Pro‐urban inequalities were perceived in terms of both outcome variables. This study reported that children from rural areas had relatively higher U5MR than children from urban areas, which is consistent with a previous study conducted in sub‐Saharan Africa.^60^ One of the possible reasons behind the lower mortality in urban areas could be the more availability of skillful and trained healthcare providers and higher knowledge and practices of healthcare seeking among urban women compared to rural women.^61^ In addition, the proportion of home delivery is higher among rural women, which may increase their risk of delivery‐related complications.^58^ Similarly, urban mothers are more likely to seek care for pneumonia symptoms for their children than rural mothers. This finding could be attributed to lower knowledge of when and where to seek care and lower education and awareness among rural women, which mostly affect their healthcare‐seeking for their offspring.^61^ Nevertheless, higher poverty in the rural areas of Bangladesh can be another barrier to getting healthcare, yielding a lower prevalence of healthcare seeking for pneumonia by rural mothers.^62^

Regarding U5M, Dhaka and Khulna divisions consistently showed better situations, with Sylhet having the worst. On the contrary, in the case of healthcare‐seeking behavior for pneumonia, no consistent trend in the prevalence was observed among the subnational regions. This indicates further widespread prospective research is warranted among a large sample to estimate the inequalities in health care seeking for pneumonia in U5C across administrative divisions in Bangladesh and identify the causal reasons and risk factors of these inequalities. The plausible reason behind the disparities in U5MR among the subnational regions could be due to the fact that women from Khulna division belong to the higher wealth quintile and attain higher education,^63^ both of which are associated with lower U5M. On the other hand, the women from Dhaka division may have access to better health facilities and quality healthcare providers. However, women from the Sylhet division were found to have lower access to health facilities and a higher prevalence of home delivery, leaving them vulnerable to higher birth infections, resulting in a higher prevalence of under‐five deaths.^64^

### 4.1 Strengths and limitations

Magnitude and trend in the U5MR and healthcare‐seeking behavior among the mothers for pneumonia, the leading cause of U5M in Bangladesh, were examined over time in this study. Measuring inequalities in these two outcomes at a time gave a better understanding of the trend and magnitude that may help policymakers design a comprehensive action plan to address these inequalities. We have used nationally representative BDHS survey data, which makes the findings of our study generalizable to the whole population. Besides, to measure the inequality, we used HEAT software by WHO and measured both absolute and relative inequality indices on both socioeconomic and geographic domains. The intuitive analytical technique allowed us to compare the findings better, which eventually strengthened this study's quality. Nevertheless, our study is not free from limitations. Since the secondary data were collected using a cross‐sectional design, we could not establish any temporal relationship or find the cause of inequality for any outcome variables. Besides, this specific analytical technique limited us in choosing other equity dimensions since limited variables are available in the WHO HEAT software.

### 4.2 Policy recommendations

Policies should be designed to reduce the disparities in U5MR and healthcare‐seeking behavior for pneumonia. Comprehensive health education and awareness campaigns are required, according to the observed educational inequality in the behavior of individuals seeking healthcare for pneumonia. The goal of these initiatives should be raising awareness among mothers with lower levels of education about the significance of obtaining healthcare for pneumonia and other children's diseases as soon as possible. The presence of gender‐related differences in U5MR suggests that gender‐sensitive health policies and initiatives are required. These ought to target the increased mortality risk among male youngsters under five. Special attention should be given through policies and programs to the high‐risk male U5C. The results of the study indicate that improving the socioeconomic status of women could contribute to declining inequalities in U5MR and healthcare‐seeking behavior for pneumonia. Thus, policies should work to empower women both socially and economically. Examples of this include enacting social protection programs for underprivileged women and advocating for equal access to work and education. Policies have to focus on expanding underprivileged populations' access to quality healthcare, including those living in rural areas and the Sylhet division. This might be accomplished through funding mobile health clinics, hiring and educating healthcare personnel, and making investments in the infrastructure of healthcare facilities.

## 5 CONCLUSIONS

This study showed that U5M decreased and the prevalence of healthcare‐seeking behavior for pneumonia among U5C increased; however, both socioeconomic and geographical inequalities remain. For both U5M and healthcare‐seeking behavior for pneumonia, wealthier subgroups, urban residents, and higher‐educated women were found advantageous. To address these inequalities to enhance healthcare‐seeking for pneumonia among U5C, and to reduce childhood mortality, the causes of these inequalities must be identified. Hence further longitudinal studies are warranted. Moreover, appropriate policies and priority‐specific measures should be taken, focusing on the disadvantaged subgroups. To make sure that socially disadvantaged subpopulations are not left behind, it is also advised to prioritize women of children from disadvantaged regions, those with poor healthcare‐seeking behavior, and those with higher U5MRs.

## AUTHOR CONTRIBUTIONS

**Satyajit Kundu**: Conceptualization; data curation; formal analysis; investigation; methodology; software; supervision; validation; visualization; writing—original draft; writing—review & editing. **Md Wahidur Rahman Nizum**: Writing—original draft. **Fahmida Fayeza**: Writing—original draft. **Syed Sharaf Ahmed Chowdhury**: Data curation; writing—original draft. **Jhantu Bakchi**: Writing—original draft. **Azaz Bin Sharif**: Investigation; supervision; validation; writing—original draft; writing—review & editing.

## CONFLICT OF INTEREST STATEMENT

The authors declare no conflict of interest.

## TRANSPARENCY STATEMENT

The lead author Satyajit Kundu affirms that this manuscript is an honest, accurate, and transparent account of the study being reported; that no important aspects of the study have been omitted; and that any discrepancies from the study as planned (and, if relevant, registered) have been explained.

## Data Availability

The study used data from the 2017–2018 Bangladesh Demographic and Health Survey. The data set is available at: https://dhsprogram.com/data/available-datasets.cfm. SK had full access to all of the data in this study and takes complete responsibility for the integrity of the data and the accuracy of the data analysis.
